# Timing and Duration of Drought Differentially Affect Growth and Yield Components Among Sugarcane Genotypes

**DOI:** 10.3390/plants14050796

**Published:** 2025-03-04

**Authors:** Amarawan Tippayawat, Sanun Jogloy, Nimitr Vorasoot, Nakorn Jongrungklang, Collins A. Kimbeng, John L. Jifon, Jidapa Khonghintaisong, Patcharin Songsri

**Affiliations:** 1 Department of Agronomy, Faculty of Agriculture, Khon Kaen University, Khon Kaen 40002, Thailand; tipamarawan@gmail.com (A.T.); sjogloy@gmail.com (S.J.); nimitr1945@gmail.com (N.V.); nuntawootjrk@gmail.com (N.J.); jidapa.k.h.thais@gmail.com (J.K.); 2 Department of Agriculture, Khon Kaen Field Crops Research Center, Khon Kaen 40000, Thailand; 3 Northeast Thailand Cane and Sugar Research Center, Khon Kaen University, Khon Kaen 40002, Thailand; 4 Sugar Research Station, Louisiana State University Agricultural Center, St. Gabriel, LA 70776, USA; ckimbeng@agcenter.lsu.edu; 5 School of Agricultural, Earth and Environmental Sciences, University of KwaZulu-Natal, Pietermaritzburg 3201, South Africa; 6 Texas A&M AgriLife Research Center, Texas A&M University System, Weslaco, TX 77840, USA; john.jifon@ag.tamu.edu; 7 Center of Knowledge and Technology for Cane and Sugar, Chatuchak, Bangkok 10900, Thailand

**Keywords:** water deficit stress, biomass partitioning, stress resistance, irrigation, crop growth rate, height growth rate, inter-specific hybrids

## Abstract

Drought significantly impacts sugarcane yield, making drought resistance an important trait in drought-prone regions. The effects of the timing and duration of drought on yield and yield components, including relationships among these traits, were examined using a diverse set of sugarcane genotypes in a 2-year (planted cane and first ratoon) field study. Three drought treatments (no water stress (SD0), short-term (SD1), and long-term (SD2) drought) were assigned as the main plot and replicated four times. Within each plot, six genotypes were nested in a split-plot design. Drought reduced yield and its components, with the decline greater in SD2 than in SD1. Strong relationships between yield and its components like stalk height and density and height growth rate, especially under drought, make these traits potential surrogates for yield in drought screening experiments. The genotypes F03–362 and KK3 displayed high, stable yield potential across drought treatments, but KK3 lost potential in ratoon crop under drought. Although KK09–0358 displayed high yield potential, it was very sensitive to drought stress while UT12 and KK09–0939 displayed low yield potential and sensitivity to drought. TPJ04–768 displayed low but stable yield potential across drought treatments and crops. F03–362 and TPJ04–768 have utility in studies seeking to couple physiological with agronomic parameters promoting drought resistance and as parents for developing cultivars combining high and stable yield performance under drought.

## 1. Introduction

Sugarcane (*Saccharum* spp.), a tall perennial C_4_ grass, is one of the most efficient species in assimilating CO_2_ and light for conversion into biomass and accounts for over 75% of the sugar produced globally [1,2]. Sugarcane production plays a crucial role in the socioeconomic development of many communities by generating employment and export earnings. Besides sugar production, sugarcane is also an excellent feedstock for renewable bioproducts and provides other ecosystem functions such as providing wildlife habitat [2,3,4,5]. It is widely cultivated in tropical and subtropical regions of the world such as Brazil, India, China, and Thailand, under rainfed farming systems. However, insufficient, erratic, and unreliable rainfall patterns often limit production potential unless supplemental irrigation is provided. Recurring drought poses a major challenge to the further expansion of sugarcane production in many of these regions, such as Thailand which is currently ranked fourth in sugarcane production and second in sugar exports globally [6,7]. In Thailand, sugarcane is traditionally planted during the early rainy season months (April to June), and late rainy to dry season months (October to February). For the late rainy season planting, the crop mostly depends on residual soil moisture at the early growth stage. Hence, the late-season crop usually experiences drought periods during its early developmental stages, which can last for a short or long durations depending on the amount of rainfall received by the end of the preceding rainy season [7,8,9,10], and this can severely reduce the sugarcane yield [1,11].

Several studies have documented the effects of drought on primary physiological and biochemical processes that ultimately determine yield [12,13,14,15,16,17]. Drought stress accounted for up to a 60% reduction in sugarcane yield [18]. The severity of yield reduction often depends on inherent variation among cultivars in physiological, biochemical, morphological, and other agronomic traits, which allow the crop to tolerate stress without significantly impacting productivity. In many cases, the important resistance traits depend on whether they are dynamic, exhibiting plasticity in response to predictable environmental stressors, or static and unresponsive to seasonal environmental cues [19]. A better understanding of how yield components of sugarcane respond to drought at different growth stages will facilitate the development of resilient cultivars and management practices to sustain productivity in a rapidly changing environment.

The rate of biomass accumulation, commonly expressed as the crop growth rate (CGR), is an integrating parameter used to estimate overall genotype performance under a given set of environmental conditions [20]. Due to its sensitivity to stresses such as drought at different phenological stages, it has been used in breeding programs to assess yield potential among cultivars under diverse conditions [21]. The CGR increased for up to 330 days after planting (DAP), after which the rate of increase declined [22]. Components of the CGR such as leaf elongation and height growth rates have also been shown to be extremely sensitive to drought [23,24]. Under drought stress, the CGR and stem elongation rates measured between 150 and 210 DAP in planted cane crop were comparatively lower than under no stress [25]. Because the ratoon crop generally has a shorter duration, the effects of drought may be different than in the planted cane crop. The planted cane is generally harvested between 13 and 15 months after planting (MAP), when it is thought to have attained its peak in sugar accumulation [26]; the subsequent ratoon crops are harvested at 10–12 months after harvest (MAH) [27]. Khonghintaisong et al. (2021) [28] provided a comprehensive study on the effects of drought on sugarcane in both the planted and first ratoon cane crops. In the study, two drought treatments, field capacity and rainfed treatment, were used, which considered the relative effects of drought stress but not the duration of the drought treatment.

The objectives of the current study are as follows: (1) to characterize the effects of the timing and duration of drought on growth, yield, and yield components among a potential set of sugarcane genotypes, (2) determine the relationships among yield, crop growth rate (CGR), height growth rate (HGR), and yield components in plants experiencing drought during critical developmental phases, and (3) detect genotypic differences in drought resistance and trait behavior that confer resistance among a diverse set of sugarcane genotypes under drought conditions. This study used commercial cultivars as well as hybrids derived from crosses between commercial cultivars and *S. spontaneum*, a wild relative of sugarcane and a known source of fitness alleles for introgression into cultivated sugarcane.

## 2. Results

### 2.1. Meteorological Conditions, Soil Moisture Contents, and Plant Water Status

The total amount of water received (rainfall + irrigation) for the three drought treatments in the planted cane crop were 984.98 mm for SD0 *=* no water or drought stress; 627.63 mm for SD1 = short-term drought for 3 months during tillering to stem elongation and early grand growth phases; and 481.24 mm for SD2 = long-term drought for 5 months during pass of the establishment, tillering to stem elongation, and early grand growth phases in the dry period (first 6 months). The corresponding water received (rainfall + irrigation) in the ratoon crop was 975.33 mm, 601.37 mm, and 443.95 mm, respectively, for the SD0, SD1, and SD2 treatments. During the recovery period, the SD1 and SD2 treatments were provided water up to field capacity (FC level). Thus, the total amounts of water received (rainfall + irrigation) by 12 MAT/MAH in the SD0, SD1, and SD2 treatments were 1847.28 mm, 1489.93 mm and 1343.54 mm, respectively, for the planted cane and 2163.03 mm, 1789.07 mm and 1631.65 mm, respectively, for the ratoon cane.

**Figure 1:**
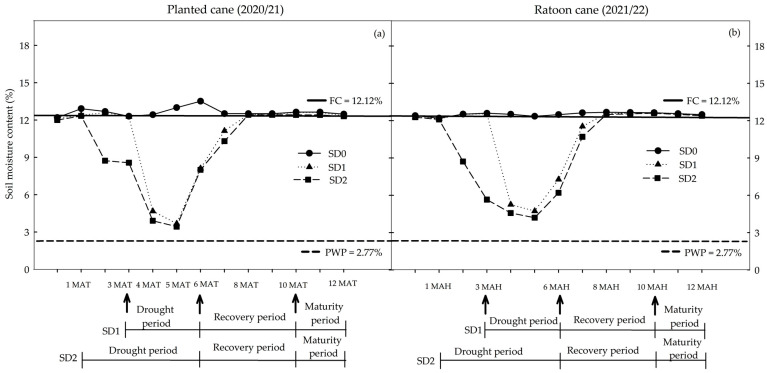
Soil moisture content (SMC) at 0–60 cm below the soil surface at 0, 1, 2, 3, 4, 5, 6, 7, 8, 9, 10, 11, and 12 months after transplanting (MAT) in the planted cane 2020/21 (**a**) and months after harvesting (MAH) in the ratoon cane 2021/22 (**b**) averaged across six sugarcane genotypes subjected to no water stress (SD0), short-term water treatment (SD1), and long-term water treatment (SD2). FC = field capacity; PWP = permanent wilting point.

The average air temperature (minimum–maximum), relative humidity, and solar radiation during each growing season were 22.1–33.2 °C, 72.2%, and 18.4 MJ m*^−^*^2^ day^−1^, respectively, for the planted cane crop (Figure S1a) and 22.0–32.6 °C, 73.9%, and17.7 MJ m^−2^ day^−1^, respectively, for the ratoon crop (Figure S1b). The average soil moisture content (SMC) for the SD0 treatments was 12.1 ± 1.0% and was very similar to the field capacity for both crops (Figure 1). Within one month of initiating water deficit treatments, the average SMC values of the SD2 treatments in both crops declined significantly (~6%) relative to the SD0 treatments. Reductions in SMC were slightly greater in the SD2 treatments than in SD1. The average SMC for the SD1 treatments (with water deficit starting at 3 to 6 MAT) was 5.79% in the 4th month, decreasing to 4.43% in the 5th month, and then increasing to 8.67% in the 6th month because of rainfall. During the recovery period, the SMC of the SD1 treatments increased to FC levels by the 8th month and remained at this level until harvest (Figure 1a). In the ratoon cane, the corresponding SMC for the SD1 treatment was 5.26% in the 4th month, decreasing to 4.74% in the 5th before increasing to 7.28% in the 6th month (Figure 1b). The average SMC of SD2 treatments (five months of water deficit stress) during the planted cane crop declined from 8.73% in the 2nd month to 8.47% in the 3rd, 4.49% in the 4th, and 4.03% in the 5th before increasing to 8.14% in the 6th month because of rainfall (Figure 1a). The corresponding SMC in the ratoon cane was 8.72% in the 2nd month, dropping to 5.66% in the 3rd, 4.58% in the 4th, and 4.21% in the 5th; it then increased after the return of rain to 6.21% in the 6th month and finally returning to FC levels between the 8th month and harvest (Figure 1b).

The leaf relative water contents (RWC), averaged across genotypes, differed significantly (*p* < 0.05) among drought treatments, starting at 4 and 5 MAT in the planted cane crop (Figure S2a) and at 3 and 6 MAH for the ratoon crop (Figure S2b). In the planted cane crop, the decline in RWC for both SD1 and SD2 was evident (approx. 8%) within one month of initiating water deficit stress (4 MAT). In the ratoon crop, the drought-induced decline in RWC was significantly less severe in SD1 (approx. 1%), compared to SD2 treatments (approx. 5%). The relative decline in RWC in the ratoon crop was much smaller, compared to the decline observed during the planted cane season. However, significant differences among water stress treatments were evident between 3 MAH and 6 MAH (Figure S2b). The lowest average RWC recorded in the SD1 and SD2 treatments in planted cane was approx. 89% (at 5 MAT, Figure S2a), whereas in the ratoon crop, it was approx. 93% (at 3 MAH; Figure S2b). The recovery of RWC after drought treatments in both the planted cane and ratoon crops was completed by 6 MAT and 8 MAH, respectively.

**Figure 2:**
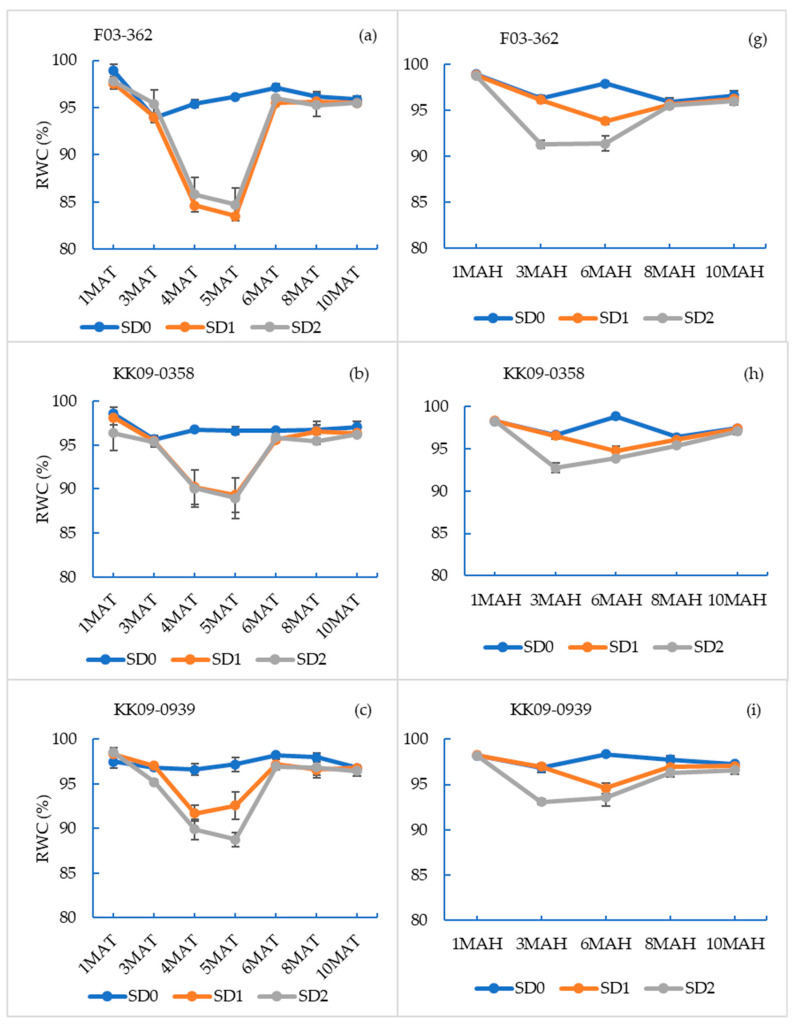

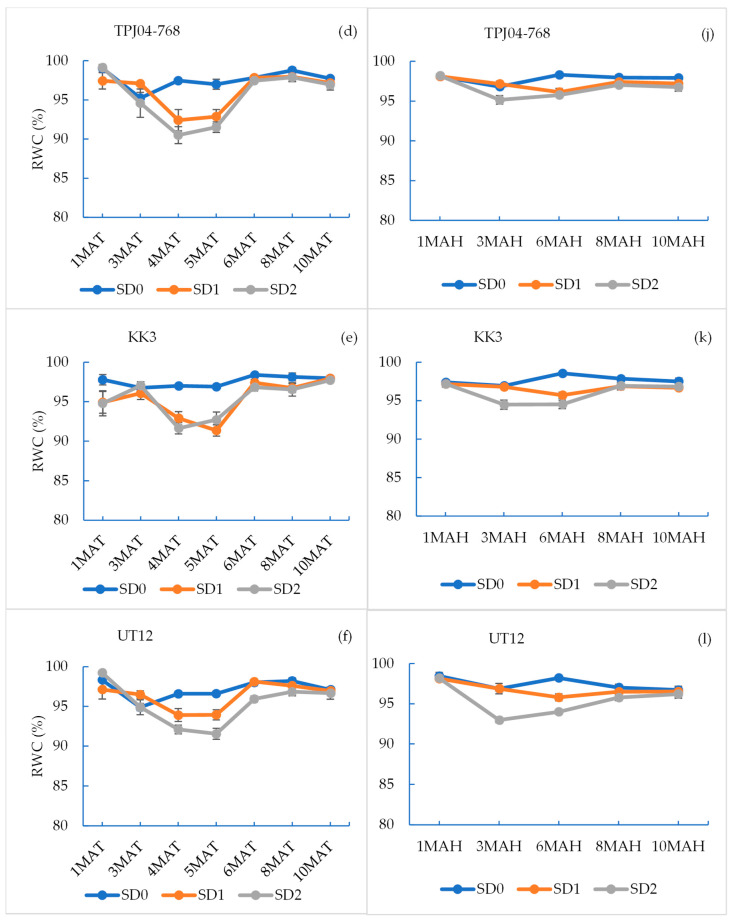
Leaf relative water content (RWC) of sugarcane genotypes sampled at 1, 3, 4, 5, 6, 8, and 10 months after transplanting (MAT) (planted cane (**a**–**f**)) and at 1, 3, 6, 8, and 10 months after harvesting (MAH) (ratoon cane (**g**–**l**)) when grown under three drought treatments. The drought treatments were no water stress (SD0), short-term drought by withholding water at 3–6 MAT/MAH (SD1), and long-term drought by withholding water at 1–6 MAT/MAH (SD2). Sampling times are on the x-axis. Values reported are mean ± SE (n = 24).

The relative water contents for the individual genotypes over the sampling periods are shown in Figure 2 (a–f; planted crop and g–l; ratoon cane), with the general profile closely mirroring the results reported in Figure S2a,b. However, the different genotypes differed in the extent to which drought affected the decline and recovery of RWC during the drought and recovery periods, indicating differences in response patterns or mechanisms. The most dramatic response was recorded by F03–362, where the RWC dropped by over 10% in both the SD1 and SD2 treatments in both crops during the drought periods and then recovered to full capacity. This may indicate the ability of this genotype to sense drought and close its stomata to prevent excess water loss. Otherwise, the drop in the RWC values during the drought months and the rise during the recovery period did not deviate much from the overall average of the rest of the other genotypes. However, the lowest drop in RWC occurred in UT12, and this genotype was the only one with a significantly lower RWC in the SD2 treatment during the recovery period at 6 MAT (Figure 2f).

### 2.2. Drought Effects on Cane Yield and Biomass Allocation Among Tissues

**Figure 3:**
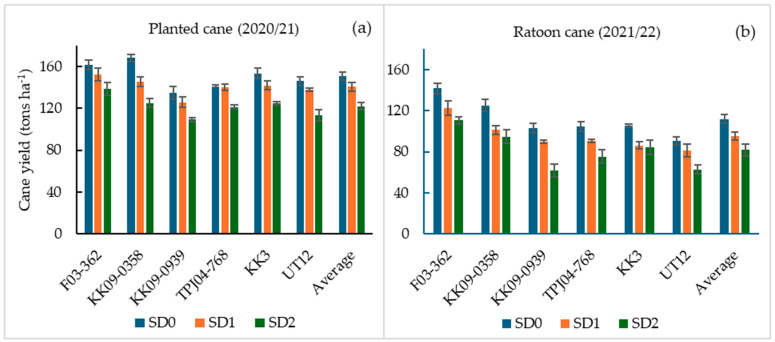
Cane yield (tons ha^−1^) at 12 MAT (**a**) and 12 MAH (**b**) of six sugarcane genotypes (x-axis) under three water treatments as SD0 (no water stress), SD1 (short-term water stress), and SD2 (long-term water stress) in the planted cane and the ratoon cane.

Average cane yields ranged from 61.9 to 168.4 tons ha^−1^ and differed significantly between the two crop years (*p* < 0.05; Table 1; Figure 3). Crop year (Y) had the most significant effect on cane yields (*p* < 0.01), accounting for 52.8% of the total variation in yield. Average cane yields recorded in the planted cane year ranged from 109.3 tons ha^−1^ (KK09–0939 under SD2 treatments) to 168.4 tons ha^−1^ (KK09–0358 under control condition, SD0) (Figure 3a). In the ratoon year, average cane yields ranged from 61.9 tons ha^−1^ (KK09–0939 under SD2 treatments) to 141.7 tons ha^−1^ (F03–362, SD0) (Figure 3b). The relative yield difference between the two crop years was lowest in F03–362, SD0 (only12.4% decline from planted cane to ratoon cane) and highest in UT12 (44.5%). Relative to the SD0 treatment, the genotypes that suffered the greatest reduction in cane yield following drought treatments were KK 09–0939 and UT 12 in both crop years (Figure 3).

The main effects of water supply (W) and genotype (G) on cane yield were highly significant (*p* < 0.01). However, the W × G and Y × W × G interaction effects were not significant (*p* > 0.05). Cane yield differences among genotypes were significant (*p* < 0.01; Table 1), and more prominent during the ratoon crop year than in the planted cane year (Figure 3). Under control conditions (SD0 treatment), the genotypes KK09–0358 and F03–362 had the highest cane yields in both years, while the lowest cane yield in both years was recorded by UT12 (Figure 3). Under short-term drought treatments (SD1), the highest cane yields were produced by F03–362, followed by KK09–0358, TPJ04–768, and KK3 in the planted cane year (Figure 3a), while the lowest yields were observed in KK09–0939 (planted cane), KK3, and UT12 (ratoon cane). The cane yield trends among genotypes under the long-term drought conditions (SD2 treatment) of planted cane were similar to those observed under SD1; however, in the ratoon year, UT12 and KK09–0939 in SD2 had the lowest overall yields (Figure 3b). Overall, F03–362, and in most cases KK09–0358, consistently produced significantly more biomass than the other genotypes under the three water supply regimes and crop years.

**Figure 4:**
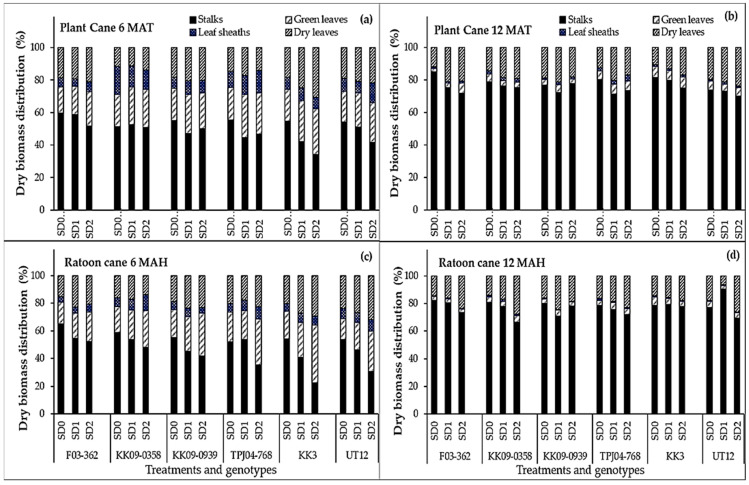
Total biomass partitioning among sugarcane varieties as a function of drought duration. Drought durations include SD0 (no water stress), SD1 (short-term water treatment), and SD2 (long-term treatment). Shown are dry biomass distribution values (%) at 6 MAT (**a**), 12 MAT (**b**) months after transplanting (top panel, planted cane) or 6 MAH (**c**), 12 MAH (**d**) months after harvesting (ratoon cane), both corresponding to the end of the drought exposure period.

At the end of drought treatments in both years (6 MAT/MAH), significant drought, genotype, and year effects on the biomass distribution among aboveground tissues (stalks, leaves, and leaf sheaths) were revealed (Figure 4). At 6 MAT/MAH, the fraction of total biomass accounted for by stalks averaged approximately 50% in the planted cane and 48% in the ratoon cane, while leaves and leaf sheaths made up the remainder. Among the genotypes, F03–362 generally had the highest stalk fractions (average of 59% in the planted cane, and 65% in the ratoon cane) while KK3 and UT12 had the lowest (average of 22 to 30%). Drought effects on biomass distribution varied among genotypes, with the decline in stalk biomass fraction (from SD0 to SD1 to SD2) showing strong linear patterns in UT12, KK3, and TPJ04–768, especially in the ratoon year (Figure 4c). At final harvest (12 MAT/MAH), the fraction of total biomass accounted for by stalks averaged approximately 76% in the planted cane, and 77% in the ratoon cane. The increase in stalk biomass allocation at 12 MAT/MAH seemed to occur at the expense of green leaf fractions, which declined from 9.2% and 6.7% (planted cane and ratoon cane, respectively) at 6 MAT/MAH to 4.9% and 3.9% (planted cane and ratoon cane, respectively) at 12 MAT/MAH. For the genotype F03–362 under SD0, planted cane again displayed the highest stalk biomass allocation fraction (approx. 85%), while for UT12 under SD2, planted cane had the lowest stalk allocation (approx. 70.2%).

**Figure 5:**
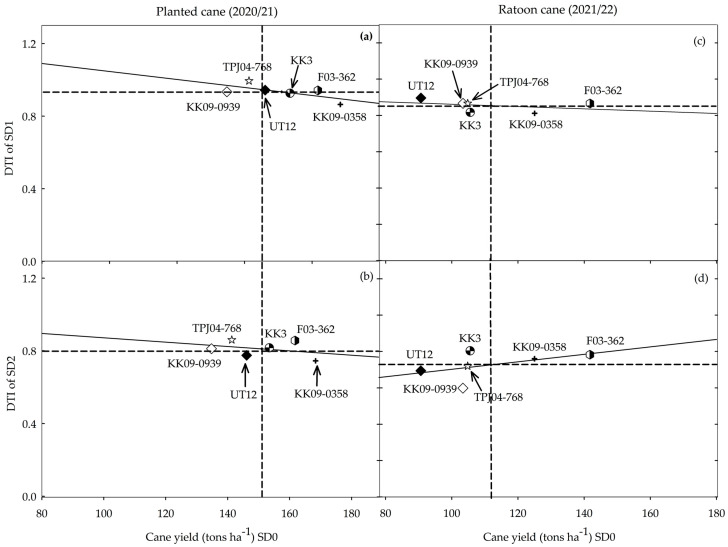
Drought tolerance index (DTI) and cane yield potential (data of non-stress, SD0) of six sugarcane genotypes subjected to two drought treatments and data collected in the planted cane (**a**,**b**) and ratoon cane (**c**,**d**) crops. SD1 (**a**,**c**) and SD2 (**b**,**d**) represent short- and long-term water treatment, respectively. Dashed lines represent the average performance while the bold line displays the relationship between the two traits in each drought treatment/crop combination.

A plot of the DTI for each genotype under the two drought regimes (SD1 and SD2) and their performances under the non-stress environment (Figure 5) displays the potential of each genotype relative to its performance under drought stress. F03–362 was consistent (Figure 5a–d) in displaying both good yield potential and drought resilience, while among the cultivars, KK 3 displayed both characteristics in the planted cane but not in the ratoon crop. Although TPJ04–768 had low yield potential, its performance was relatively stable across drought treatments and crop years, indicating its potential to be exploited in drought-prone environments where yield stability is valued. Yield potential was high in KK09–0358 but generally declined under drought conditions, while UT 12 and KK09–0939 were generally lacking in both yield potential and stability of performance under drought conditions.

### 2.3. Drought Effects on Yield Components and Growth Rate (Height Growth Rate (HGR), Crop Growth Rate (CGR)) Among Genotypes

In a sugarcane production system, cane yield generally consists of the number of stalks and the size of the stalks, which also determines the weight per stalk. Therefore, the relationship among these characteristics within individual genotypes subjected to different water regimes could provide greater insights into a genotype’s trait dynamics while under water stress. Cane yields at 12 MAT/MAH showed strong correlations with several yield components measured just after cessation of the drought treatments (6 MAT/MAH; Table 2). Average leaf area index, millable stalk density, stalk height, stalk elongation rate or height growth rate (HGR), and crop growth rates (CGR) all showed strong positive correlations (r values > 0.468) with cane yield assessed at 12 MAT/MAH. Correlations between cane yield and leaf relative water contents (RWC) and stalk diameter were generally small and non-significant. In both crop years, correlations between cane yield and HGR were much stronger (r values > 0.8) than those between cane yield and CGR (r values > 0.7). Stalk height, internode lengths, and stalk elongation rates displayed strong and significant positive correlations. However, stalk height, stalk density, internode lengths, and stalk elongation rates all showed strong negative correlations with stalk diameter (Table 2).

**Figure 6:**
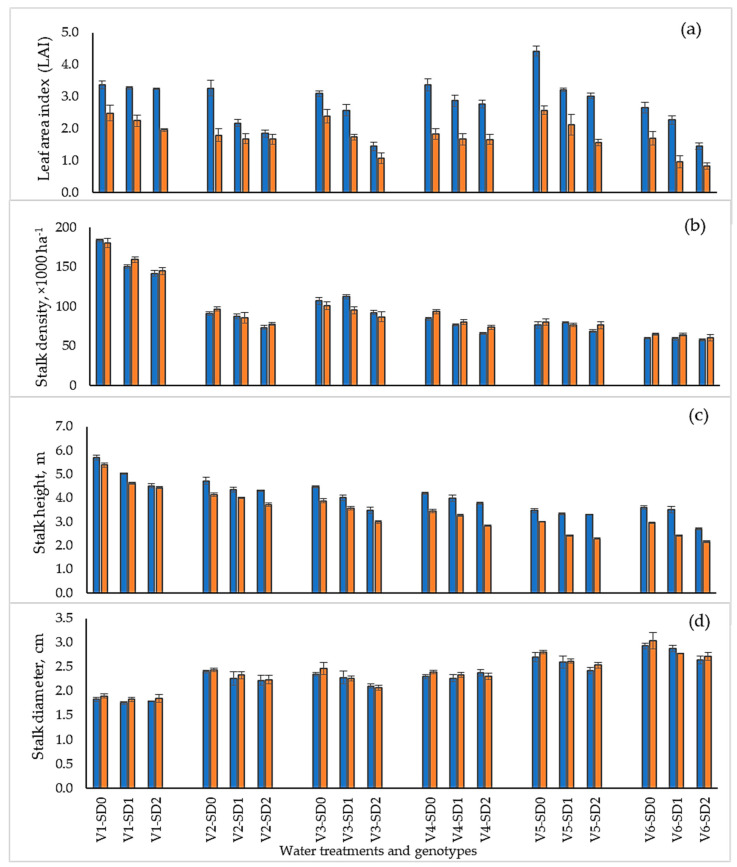
Leaf area index (**a**), millable stalk density (**b**), stalk height (**c**), and stalk diameter (**d**) of sugarcane genotypes after growth under three drought treatments for planted cane (2020/21; blue bars) and ratoon cane (2021/22; orange bars) crops. Drought treatments were SD0 (no water stress), SD1 (short-term drought for 3 months during tillering to stem elongation and early grand growth phases), and SD2 (extended water stress, long-term drought for 5 months during pass of the establishment, tillering to stem elongation, and early grand growth phases). Genotypes: F03–362 (V1), KK09–0358 (V2), KK09–0939 (V3), TPJ04–768 (V4), KK3 (V5), and UT12 (V6), respectively, at 12 MAT/MAH.

The leaf area index (LAI) at 12 MAT/MAH was significantly higher in the planted cane year (ranging from 1.5 to 4.4) than the ratoon cane year (range 0.8 to 2.6; Figure 6a). The genotype KK3 had the highest average LAI values, especially during the planted cane under control (SD0) conditions. Drought exposure strongly reduced the LAI values of all genotypes except F03–362 (Figure 6a).

Millable stalk densities ranged from 57.6 to 183.4 thousand per hectare and differed only slightly between the two crop years. At final harvest (12 MAT/MAH), the genotype F03-362 had nearly twice the number of stalks per hectare compared to all the other genotypes (Figure 6b). Drought effects on millable stalk densities were significant and followed similar trends for all genotypes.

**Figure 7:**
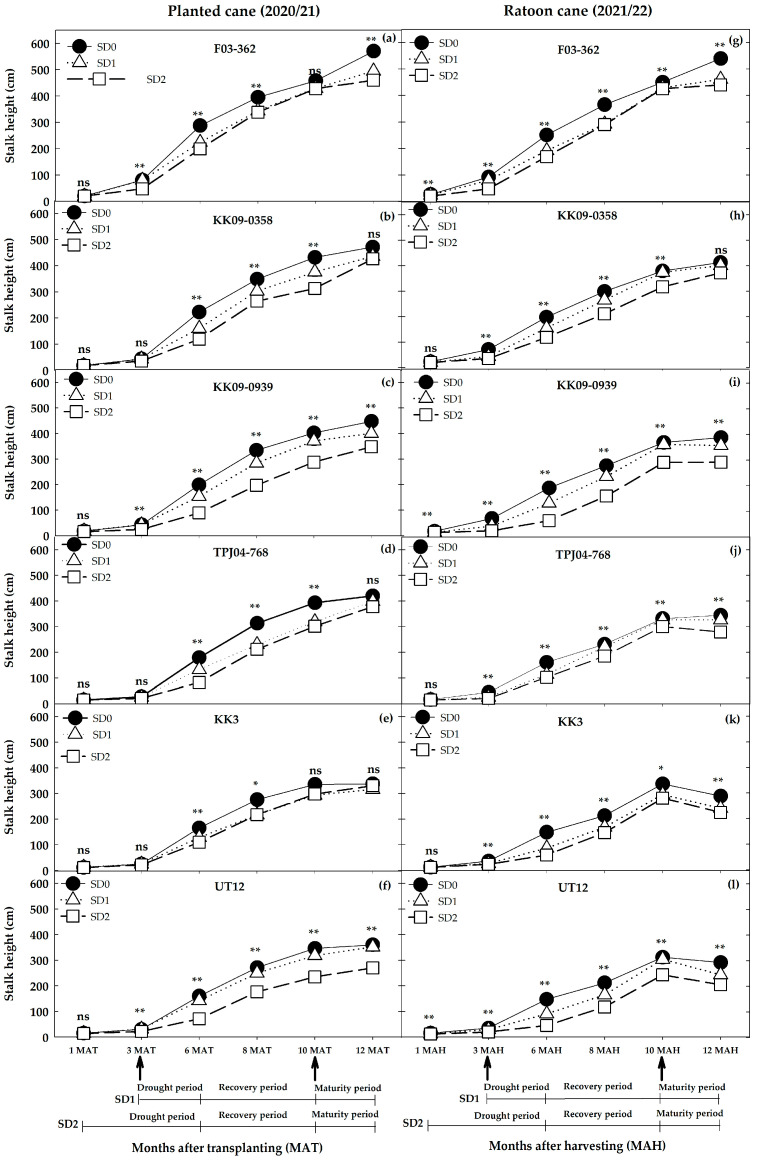
Mean stalk height (cm) of six genotypes grown for two seasons (planted cane 2020/21 and the ratoon cane 2021/22) under three water treatments (SD0 = no water stress, SD1 = short–term water treatment at 3–6 months after transplanting (MAT) in the plant cane and short–term water treatment at 3–6 months after harvesting (MAH) in the ratoon cane, SD2 = long–term water treatment at 1–6 MAT/MAH). Water was reintroduced during the recovery period (6–10 MAT/MAH) while the maturity period (10–12 MAT/MAH) is a phenological stage when sugarcane undergoes ripening. ns, *, and ** indicate non-significant, significant, and highly significant by least significance difference (LSD) method at *p* ≤ 0.05 and *p* ≤ 0.01 probability levels, respectively.

Variation among genotypes in stalk height showed a similar trend to stalk density (Figure 6c). The average millable stalk heights ranged from 2.7 to 5.7 m in the planted cane year and from 2.2 to 5.4 m in the ratoon crop. Differences in stalk height between the planted cane and ratoon crop for each water treatment were stronger in UT12, KK3, and TPJ04–768 compared to KK09–0939, KK09–0358, and F03–362. Height elongation rates (HGR) differed significantly between crop years, water treatments, and among genotypes in nearly all the measurement periods, as indicated by the significant three-way G × W × Y interaction (*p ≤* 0.01; Table 3 and Table 4). Water treatment made up a greater part of the variation during the drought periods (1–3 months, 23.4%; 3–6 months, 45.9%) compared with the recovery and physiological maturity periods. The two-way interaction between W × Y produced significantly different height growth rates during the 1–3 month period but not during 3–6 and 6–8 months. Significant differences in the HGR resumed during 8–10 months (recovery period) and 10–12 months (physiological maturity period). The height growth rate differed significantly among genotypes in all periods. Compared with other effects, the genotype main effect accounted for a disproportionately large variation during the 1–3 month (57.5%) period, with another large variation (30.8%) occurring during the physiological maturity (10–12 months) period. The G × Y interaction for the HGR contributed little to the total variation, although a significant interaction was absent only during the 8–10 month period. The G × W interaction effect for HGR was significant in all periods except at 6–8 months, while the G × W × Y effect was not significant only at 8–10 months. The effect of water treatments on the HGR in the planted cane, across all genotypes, significantly differed in this order: SD0 = SD1 > SD2 at 1–3 MAT and SD0 > SD1 > SD2 at 3–6 MAT, respectively. (Table 4). Under the drought phase, KK3, the more drought tolerant of the two cultivars, had HGR values of 0.22, 0.20, and 0.16 cm day^−1^ under SD0, SD1, and SD2, respectively, at 1–3 MAT and 1.58, 1.61, and 1.00 cm day^−1^ under SD0, SD1, and SD2, respectively, at 3–6 MAT (Table 4). During early development (1–3 MAT/MAH), the average HGRs of F03–362 were significantly higher than those of the other genotypes and only declined significantly in response to water deficits under the SD2 treatments. During the tillering period (3–6 MAT/MAH), the HGRs of F03–362 continued to increase under all water regimes. However, during the recovery periods (6–8 MAT/MAH), the superior HGR of F03–362 was maintained only under the SD1 and SD2 water regimes (Table 4; Figure 7). Thereafter, the HGRs of F03–362 declined during the (8–10 MAT/MAH), and briefly increased in the 10–12 MAT period under the SD0 and SD1 water regimes (Figure 7). For all the other genotypes, the HGRs were initially slow (compared to F03–362) and more sensitive to water deficit treatments in both the 1–3 and 3–6 MAT periods. However, during the recovery periods (6–8, 8–10, and 10–12 MAT), these genotypes displayed strong HGR recovery such that differences among water regimes declined (Table 4; Figure 7). During the ratoon crop, the HGR of all genotypes during the drought periods 1–3 MAH and 3–6 MAH declined significantly under the two water stress treatments. The genotype F03–362 had a higher HGR than the other genotypes in all treatments, although drought decreased the HGR: SD0 > SD1 > SD2 at 1–3 MAH and SD0 > SD1 = SD2 at 3–6 MAH. KK09–0358 and KK09–0939, the second-ranked genotypes, had a high HGR in the three water treatments from 1 to 3 and 3 to 6 MAH, except KK09–0939 in SD2 (Table 4). TPJ04–768, KK3, and UT12, the third-ranking genotype, had lower HGRs in the three water treatments except for KK3 in SD2 at 1–3 MAH and for TPJ04–768 in SD2 at 3–6 MAH. After the drought treatment, the HGR of each genotype decreased in the order SD0 > SD1 > SD2 at 1–3 MAH except for SD0 > SD1 = SD2 in KK3, whereas at 3–6 MAT, F03–362 and TPJ04–768 decreased in the following order: SD0 > SD1 = SD2. KK09–0358, KK09–0939, KK3, and UT12 showed decreased HGRs after being subjected to water treatments, as follows: SD0 > SD1 > SD2.

Analysis of variance of the crop growth rate (CGR, g m^−2^ day^−1^) during different periods (drought, recovery, and physiological maturity) combined for both crops (planted cane and ratoon cane crops) showed significant crop year effects (Table 5) across most measurement periods. As a percentage of the variation within a period, crop year effects were highly significant during the drought and recovery periods. The proportion of variation for the water treatment (W) effect was larger for the 3–6 month (65.6%) than the 1–3 month (22.1%) period, suggesting that the longer the drought, the greater the effect. The recovery period of 8–10 months accounted for more (40.3%) variation than the 6–8 month period. Although significant at the 1–3, 3–6, 6–8, and 10–12 month periods, the two-way interaction effect of W × Y was not significant, except during the 1–3 months period where it contributed 19.3% to the total variation. Significant differences in the CGR were also observed among genotypes across all periods, with the 1–3, 3–6, 6–8, 8–10, and 10–12 month periods contributing 18.3, 13.7, 13.6, 8.5, and 21.1%, respectively, to the total variation (Table 5).

In the planted cane during the drought period, at 1–3 and 3–6 MAT, the CGRs across the six sugarcane genotypes were significantly different between SD0 and SD2 from 1 to 3 MAT; however, significant differences in the CGR among all three water treatments were found: SD0 > SD1 > SD2 from 3 to 6 MAT (Table 6). KK3, a tolerant genotype, showed 0.061, 0.036, and 0.026 g m^−2^ day^−1^ CGR values, respectively, the under three water treatments at 1–3 MAT, and 0.221, 0.136, and 0.065 g m^−2^ day^−1^, respectively, under the three water treatments at 3–6 MAT. The CGR was the highest in the F03–362 genotype compared to other genotypes from the 1–3 MAT to 3–6 MAT; when affected by drought, the CGR was decreased in SD2 at 1–3 MAT and in SD1 and SD2 at 3–6 MAT; at 1–3 MAT, KK3 and UT12 did not show decreased CGRs, but during 3–6 MAT, the CGRs of KK3 and UT12 under SD1 and SD2 decreased. KK09–0939 and TPJ04–768 had high CGRs under SD1 and SD2 at 3–6 MAT. The CGR was affected during drought durations, and the CGR decreased for all genotypes. Four inter-specific genotypes had a higher CGR than the commercial genotypes at 3–6 MAT (Table 6).

During the recovery period, the four inter-specific genotypes had a higher CGR than the commercial genotypes in SD1 at 6–8 MAT. F03–362 had the highest CGR in SD1 and SD2, whereas KK09-0358 had a high CGR in SD1. At 8–10 MAT, the CGR values were lower under SD1 and SD2 than those of SD0. KK09–0939 and TPJ04–768 had high CGRs in SD1 and SD2, whereas TPJ04–768 had a high CGR in SD2.

**Figure 8:**
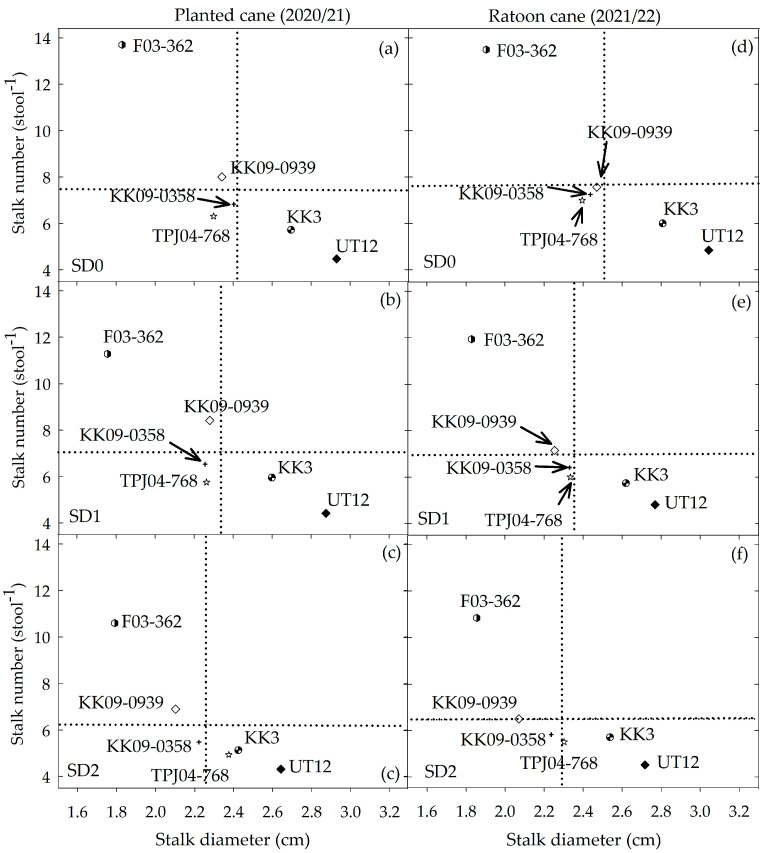
Relationship between stalk number (stool^−1^) and stalk diameter (cm) of six sugarcane genotypes in planted cane (12 MAT) and ratoon cane (12 MAH) under three drought durations under SD0 = no water stress (**a**,**d**), SD1 = short-term water treatment: 3–6 MAT/MAH (**b**,**e**), and SD2 = long-term water treatment: 1–6 MAT/MAH (**c**,**f**). Means with ns are not significant at *p* = 0.05 by least significant difference (LSD) analysis. Arrows are only used to point to specific genotypes in the figure. The vertical (stalk diameter) and horizontal (stalk number) dotted lines represent the mean of each trait averaged across genotypes.

Stalk diameter did not differ significantly between crop years or among water treatments for each genotype (Figure 6d). Among the genotypes, there was an inverse relationship between stalk diameter and all the other important yield components (LAI, stalk height, stalk density, internode length, and stalk elongation rates (Table 2)). While drought stress caused a larger decrease in stalk number compared with stalk diameter, it did not alter the relative sensitivity of this trait among the genotypes studied. The genotypes could be divided into three groups: (1) F03–362 as small stalk diameter but high stalk number; (2) KK3 and UT12 were commercial genotypes with a large stalk diameter but low stalk number; and (3) KK09–0358, TPJ04–768, and KK09–0939 (backcross of inter-specific hybrids group) were intermediate in both stalk diameter and stalk number (Figure 8).

## 3. Discussion

### 3.1. Meteorological Conditions, Soil Moisture Contents, and Plant Water Status

Drought is a major factor contributing to reduced sugarcane yields in tropical regions [29], with early-season drought encompassing both short- and long-term impacts [30]. Any drought study seeking to understand resistance mechanisms or the effects of drought on agronomic characteristics is better achieved by examining specific genotypes or genotypes important to a specific region under conditions that mimic specific targeted environments. This study examined a diverse set of genotypes under short- (SD1) and long-term (SD2) drought conditions akin to those experienced in the northeastern region of Thailand. A potential application of the results from this study is to select germplasm and surrogate traits, which are measurable characteristics that can be used to indirectly select for drought resistance and for use in developing drought-resilient varieties.

In this study, the average soil moisture contents of the control plots (SD0) were maintained within 1% of field capacity. Soil moisture contents for the simulated drought treatments (SD1 and SD2) in both the planted and first ratoon cane crops declined in SD2 from mid-December to mid-May during the early part of the establishment phase, and during the tillering to stem elongation and early grand growth phases, and in the SD1 treatment from mid-February to mid-May during tillering to stem elongation and the early grand growth phases [31]. Thereafter, the SMC slowly increased until about July (the recovery period). Thus, all genotypes experienced an adequate period of drought before recovery, as is common during the sugarcane growing seasons in Thailand. This was evident from the significant reduction in the performance of all traits in the SD1 and SD2 treatments compared to the SD0 treatment. Although many studies have documented drought effects on sugarcane productivity in Thailand [5,28,32], a considerable gap exists in the knowledge regarding drought intensity and duration impacts on critical developmental stages of sugarcane growth. This study documented the differential responses of yield and yield components to varying drought exposure durations among diverse genotypes. When water stress was imposed for a short duration (SD1) targeting the tillering growth phase, the responses of primary yield-determining processes and traits such as RWC were less severe compared to responses to long-term exposure (SD2). Even though water deficit stress treatments were imposed during the early growth stages (tillering and early grand growth phases), there were carryover effects on the final cane yields. As the frequency and duration of extreme weather events continue to increase [33], it is conceivable that commercial cane production in rainfed regions will experience drastic yield reductions, like the responses observed in this study.

According to Castro et al. (2002) [34], the detrimental effects of drought exposure on the plant crop can sometimes carry over into the ratoon crop. This depends on the intensity (severity) and duration of the drought exposure. While this was not a testable hypothesis in this study, it was possible to assess the effect of the same intensity and duration of drought in both the planted and first ratoon cane crops. Significant year effects for biomass, cane yield, and stalk height indicate that, for these traits, exposure to drought resulted in more severe adverse effects in one crop compared with the other. While conventional wisdom would indicate that the ratoon crop would suffer less from drought because it already has an established root system, in this study, the percent reduction was higher in the ratoon compared with the planted cane crop in both drought treatments. Therefore, although the plant crop had the opportunity to recover following drought exposure, it is possible that the recovery was not sustained in susceptible genotypes following further exposure to drought.

### 3.2. Drought Effects on Cane Yield and Biomass Allocation Among Tissues

Biomass was the trait most affected by the drought treatment, accounting for up to 50 percent of the total variation. The two-way interaction effect of W × Y was also significant for biomass, with the SD2 treatment accounting for the greatest impact. Biomass was reduced by 49% in the ratoon crop compared with 38% in the planted cane crop for the SD2 treatment, as opposed to 32% in the ratoon cane compared with 31% in the planted cane crop in the SD1 treatment. The results indicate that susceptible varieties experiencing prolonged exposure to drought in the planted cane crop are likely to suffer irreparable damage if they are resubmitted to prolonged periods of drought after recovery.

The biomass distribution among major aerial organs differed across water treatments and among genotypes (Figure 4). Reductions in biomass allocation to stalks (the important yield-bearing organ) in response to drought were evident and varied among genotypes. Notably, the stalk biomass allocations of KK3 and UT12 were more sensitive to drought exposure, as indicated by the strong linear declines in stalk biomass fractions under SD1 and SD2 (Figure 4). This trend was evident in both the planted and ratoon cane crops. At the onset of recovery from drought exposure (6 MAT/MAH), F03–362 had the highest stalk biomass fractions for a given water treatment in both the planted and ratoon cane crops. Biomass allocation to each component crop organ reflects the crop’s ability to acquire and utilize available essential resources and plays a critical role in the crop’s ability to cope with a dynamic growing environment characterized by multiple stress factors. Many investigations have demonstrated that under drought stress, many plants respond by adjusting their assimilate distribution patterns, notably with increased preferential biomass allocation to the roots to maintain the water absorption capacity while reducing allocation to above-ground organs such as stems and leaves to minimize water loss through transpiration, thereby avoiding tissue desiccation and maintaining a favorable internal water status even under drought [35,36,37]. While root responses were not documented in this study, the observed shifts in the biomass allocation patterns of the aerial organs are consistent with a strategy to conserve water and adapt to drought conditions. Further studies would help ascertain why drought-induced reductions in stalk fractions were more severe in the ratoon crop compared to the planted cane, considering that the ratoon crop already had an established root system at the beginning of the growing season. More studies will also help establish the mechanism(s) underlying the superior stalk fractions of genotypes such as F03–362.

Given that producers mostly prefer genotypes that maintain a high yield potential despite limited water availability, a plot of the DTI and yield potential is instructive in revealing genotypic differences. The genotypes could be classified in terms of their yield potential (high P/low P) when grown under no water stress and the reduction in yield potential (high R/low R) when under drought stress. Based on this metric, F03–362 (high P/low R) would be the preferred genotype that combines a high yield potential with stable (low reduction in yield potential) performance during drought stress. Whereas KK3 (high P/low R) appears to possess some level of drought resilience, this seems to break down in the ratoon crop. The other genotypes can be classified as high P/high R (KK09–0358), low P/high R (KK09–0939, UT 12), and low P/low R (TPJ04–768). Genotypes like F03–362 could be exploited in regions where rain is frequent but would also perform well during drought periods. TPJ04–768 could be exploited in drought-prone environments where yield stability is valued but, unlike F03–362, which possesses both yield potential and stability, it would fail to take advantage of incidental rainfall. More importantly, F03–362 and TPJ04–768 could also be used as parents in crosses with commercial cultivars to generate populations segregating for high P/low R.

### 3.3. Drought Effects on Yield Components, Growth Rate, and Resilience Among Genotypes

Several traits that have been linked to sugarcane productivity, namely, leaf area index, millable stalk density, stalk height, internode length, and stalk elongation rates, were strongly correlated with cane yields, and their response patterns to water deficits varied among genotypes (Table 2; Figure 6). Notably, treatment effects on stalk height at the end of the drought stress treatments (6 MAT/MAH) showed a significant three-way interaction between genotype, water treatment, and crop year, whereby the sensitivity of stalk height to water deficit was stronger in the ratoon crop than the planted cane in genotypes like KK3 and UT12, compared to others like F03–362.

Knowing how drought, especially how different durations of drought, affect cane yield and cane yield components, including growth rates, is vitally important for developing sugarcane genotypes for drought-prone environments. A substantial proportion of the total variation in biomass, stalk height, stalk diameter, stalk number per stool, and cane yield was contributed by the difference in drought duration. Similarly, results where drought was imposed at the early growth stage under rainfed conditions also reported a reduction in the stalk dry weight [28,30]. Three genotypes (KK3, UT13, and Kps01–12) have previously been classified as water deficit-tolerant cultivars based on their DTI values, which were 0.57, 0.51, and 0.71, respectively [28]. The disparity between that study and the current observations might be due to differences in the drought severity, drought duration, and weather conditions present in these two studies, which may contribute disproportionately to these crop parameters. The current study demonstrated that irrigation management is very vital for enhancing the growth rate and cane yield when sugarcane is grown in areas prone to early drought.

These results reveal that the sugarcane genotypes responded differently to the different drought treatments (drought duration) and crops years for most of the traits, with only a few exceptions. Although the interaction effects were significant for most traits, they constituted rather a small portion of the total variation when compared to the main effects (drought duration and genotype). The contribution of year effects to the total variation was small for stalk diameter and stalk number but proportionately larger for cane yield, biomass, and stalk height. This indicates that performance of these traits (stalk diameter and stalk number) was relatively consistent between years. Biomass, stalk height, and cane yield were higher in the planted cane (2020/21) than in the ratoon cane (2021/22) crop. Similar results were reported by Khonghintaisong et al. (2021) [28], who found that the average stalk dry weight of six sugarcane cultivars was 41.81–55.81% higher in the planted cane than in the ratoon season. Both results corroborate previous reports that the yield of sugarcane in Thailand is 22–36% lower in the ratoon crop compared to the planted cane [38]. Therefore, in the presence of added stress such as drought, the ratoon crop could suffer a greater reduction in performance compared to the planted cane crop. Based on these observations in cane yield, the current list of genotypes could be classified into four groups based on their cane yield potential: (1) high potential, namely F03–362 and KK09–0358; (2) medium potential, namely KK3; (3) medium-low potential, namely TPJ04–768; and (4) low potential, namely UT12 and KK09–0939.

The stalk elongation rates (HGR) and crop growth rate (CGR) increased consistently during the establishment period and attained maximum increase during the elongation growth phase. Therefore, it is not surprising that severe water deficit conditions during the early growth stage cause a significant reduction in most sugarcane traits [25]. The rate at which these traits increased slowly declines during the ripening (maturity) stage. Under normal conditions, during the maturity stage, the CGR increased continuously until about 330 days after planting (DAP) and slowly decreased after that, but, more importantly, the CGR was correlated with both the net assimilation rate (NAR) and the LAI [22]. However, as reported earlier, the CGR was higher in the planted cane (12.42 g m^−2^ day^−1^) compared to the ratoon cane (11.11 g m^−2^ day^−1^) [39]. Other traits such as NAR, maximum LAI, and total dry matter have also been found to be lower in the ratoon crop compared with the planted cane crop [39]. The peak CGR in sugarcane is about 26.0 g m^−2^ day^−1^, and this occurs during the elongation phase at about 220 DAP [21]. Sugarcane cultivars with a high CGR were noted to achieve a high cane yield [40,41]. Khonghintaisong et al. (2021) [28] found that the early water deficit reduced the leaf growth rate (LGR). Two factors including the unavailability of moisture and sugarcane plant acclimatization to improve the root/shoot ratio for maintaining water uptake are probably responsible for this reduction [42]. Khonghintaisong et al. [28] also found that during the recovery period, the height growth rate (HGR) was an important trait contributing to the stalk dry weight at final harvest, and these results were consistent in both the planted (longer water deficit duration) and ratoon (shorter water deficit duration) crops. These results are similar to ours, where the different water deficit tolerances closely mirrored the HGR and CGR. Moreover, drought- resilient genotypes, such as F03–362 and KK3, maintained higher photosynthetic activity under water deficit, exhibiting less photosynthesis decline than susceptible genotypes [30]. Under rainfed conditions, the CGR pattern during the recovery phase was a key indicator of the crop’s adaptation to both short and long water deficit periods in both plant and ratoon crops [28]. Sugarcane’s ability to recover from impacts of episodic drought stress is crucial for yield stability and is considered a key factor in drought resistance research [43]. Genotypes such as KK09-0358 that can recover from mild drought stress like SD1 and maintain a high CGR and HGR show potential for yield stability in regions with erratic rainfall.

## 4. Materials and Methods

### 4.1. Experimental Design, Plant Material, and Field Site

This two-years field study comprising a planted cane (15 November 2020 to 14 November 2021) and a ratoon cane crop (15 November 2021 to 14 November 2022) was conducted at the Khon Kaen Field Crops Research Center of Thaphra Station, Khon Kaen Province, Thailand (latitude 16°20″ N, longitude 102°49″ E, elevation 166.7 m above sea level). Six sugarcane genotypes were used in the study, including four genotypes (F03–362, KK09–0358, TPJ04–768, and KK09–0939) derived from inter-specific hybridization (commercial *Saccharum* hybrids × *S. spontaneum*), two commercial genotypes, namely Khon Kaen 3 (KK3), known to be moderately drought-tolerant, and U-thong 12 (UT12), known to be drought-susceptible [28,38,44]. The key distinguishing characteristics of these genotypes are listed in Table 7.

The experiment was established using a split-plot arrangement in a randomized complete block design with four replications. The main plot was three different water treatments: (i) control, experiencing no water or drought stress (SD0), where water was maintained at FC based on calculated evapotranspiration requirements (see Section 4.4 for more details); (ii) short-term drought treatment (SD1), where water was withheld from 3 to 6 months after transplanting (MAT) in the planted cane and 3 to 6 months after harvesting (MAH) in the first ratoon cane; and (iii) long-term drought treatment (SD2), where water was withheld from 1 to 6 MAT in the planted cane and 1 to 6 MAH in the ratoon cane crop. The six sugarcane genotypes constituted the subplots and were randomly assigned to each main plot.

### 4.2. Cultural Practices and Field Preparations

To promote uniformity among the plantlets, single eye bud sets (approx. 0.065 m in length) of each genotype were initially raised in individual plastic bags, measuring 0.000715 m^3^ (0.05 m wide by 0.13 m long by 0.11 m deep), filled with soil and filter cake mixed at 2:1 *v*/*v* and irrigated daily. After three weeks, uniform plantlets (at the 3-leaf stage) were transplanted into subplots each measuring 12 × 13 m long, with a plant spacing of 1.5 m between rows and 0.5 m between plants within a row (eight rows per plot), for a total of 72 plots (six genotypes, three main plots, and four replications). Before transplanting, holes were dug manually to a diameter of 0.20 m and a depth of 0.20 m. After transplanting, the holes were covered with soil to about 0.10 m below the soil surface. Water was applied immediately after transplanting and for about one month after using an overhead sprinkler irrigation system to aid in plant establishment. Standard cultural practices for sugarcane production in Thailand, including land preparation, fertilization, and weed control, were followed throughout the study period [46,47].

### 4.3. Experimental Conditions and Crop Management

Before transplanting, soil samples were taken from 4 representative points of each replication at 0–30 and 30–60 cm depths using an auger, and the chemical and physical properties of the soil were determined using standard methods [48] (shown in Table S1). The soil type at the experimental site is a Satuk series (Suk) fine-loamy, siliceous, sub-active, hyperthermic Typic Pale Stults [49]. The soil texture was a sandy loam with a pH of 5.35. Organic matter (OM) was 0.29%; electrical conductivity (EC) was 0.014 dS·m^−1^; available phosphorus was 51.8 mg kg^−1^; exchangeable potassium was 46.0 mg·kg^−1^; exchangeable calcium was 77.6 mg kg^−1^; exchangeable manganese was 8.4 mg kg^−1^; and the bulk density was 1.6 g cm^−3^ at 0–30 cm. The soil texture was a sandy loam with a pH of 5.83, OM of 0.13%, EC of 0.017 dS m^−1^; available phosphorus was 53.2 mg kg^−1^; exchangeable potassium was 51.6 mg kg^−1^; exchangeable calcium was 158.4 mg kg^−1^; exchangeable manganese was 15.6 mg kg^−1^; and bulk density was 1.7 g cm^−3^ at 30–60 cm.

Fertilization was based on the results from soil mineral analysis [47]. Inorganic forms of N–P_2_O_5_–K_2_O were applied at rates of 137.5 kg ha^−1^, 31.3 kg ha^−1^, and 100 kg ha^−1^, respectively. Phosphorus fertilizer was applied as a basal dose at 1 MAT/MAH, whereas nitrogen and potassium were applied in split applications, the first at 1 MAT/MAH and second at 5 MAT/MAH.

Weeds were controlled with a chloroacetanilide herbicide (Alachlor 48% *w*/*v* EC: 2–chloro–*N*–(2,6–diethylphenyl)–*N*–(methoxymethyl)acetamide) applied at pre-emergence, and hand weeding was performed as necessary. A carbamate insecticide (carbofuran, FMC AG Co., Bangkok, Thailand) was preemptively applied (2.5 L ha^−1^) in both cane and ratoon crops to control sugarcane borer (*Diatraea saccharalis*).

### 4.4. Irrigation Treatments

Plots were irrigated with water from a nearby well (pH 6.64; EC of 0.60 dS m^−1^). For control treatments (SD0), water was applied at field capacity (FC, 12.12% volumetric soil moisture content) and was maintained at this approximate level until harvest. Watering was initiated using a mini-sprinkler system after transplanting until 1 MAT/MAH, after which the mini-sprinklers were replaced with a surface drip irrigation system for the remainder of the experiment. For the SD1 treatment, water was withheld between 3 and 6 MAT (planted cane) and between 3 and 6 MAH (ratoon cane). For the SD2 treatments, water was withheld between 1 and 6 MAT/MAH. Irrigation requirements were calculated daily based on the crop water requirement (ET_crop_), as described by Doorenbos and Pruitt (1992) [50] and Khonghintaisong et al. (2021) [28]:

ET_crop_ = ET_o_ × K_c_ (1)

where ET_crop_ is the crop water requirement (mm day^−1^); ET_o_ is the evapotranspiration of a reference crop under a specified condition, calculated by the evaporation pan method; ET_o_ = K_p_ × E_pan_; K_p_ = pan coefficient (class A pan with green fetch); E_pan_ = pan evaporation (mm day^−1^); K_c_ is the crop water requirement coefficient for sugarcane at different growth stages, as follows: 0.55 at the initial stage, 0–20 day after planting (DAP); 0.80 at the establishment stage, 21–30 day after transplanting (DAT); 0.90 at the tillering stage, 31–91 DAT; 1.10 at the stem elongation stage, 92–232 DAT; 1.05 at the yield formulation or sugar accumulation stage, 233–333 DAT; and 0.60 during maturity, 334–365 DAT [28,50].

### 4.5. Data Collection

#### 4.5.1. Meteorological Data and Soil Moisture Content

Precipitation (mm), pan evaporation (mm), solar radiation (MJ m^−2^ day^−1^), relative humidity (%), and minimum–maximum temperatures (°C) were recorded by a weather station located within 100 m of the study site (Supplementary Figure S1). Soil moisture content (SMC) was also monitored and recorded gravimetrically using soil samples collected from 0 to 60 cm depths. Samples were weighed, dried (105 °C until constant weight), and reweighed. SMC was calculated as follows:

SMC (%) = [(wet soil mass − dry soil mass)/dry soil mass] × 100(2)

#### 4.5.2. Leaf Relative Water Contents

Leaf relative water content (RWC) measurements were conducted at 1, 3, 6, 8, and 10 MAT in the planted cane crop and again at 1, 3, 6, 8, and 10 MAH in the ratoon crop, following the methods previously described by Silva et al. (2013) [51]. The fully expanded leaf from the top visible dewlap was sampled between 09:00 and 11:00 a.m., and 2 cm^2^ leaf disks were taken from the top, middle, and bottom portions of the leaf blade. The leaf disks were placed immediately in a tube, kept on ice, and transported to the laboratory where the fresh weights were recorded. The leaf disk samples were placed in distilled water for 24 h after, which the leaf surfaces were blotted dry and the saturated (turgid) weights were recorded. The samples were then oven-dried at 80 °C for 48 h, and the leaf dry weights were recorded. The RWC was calculated from the equation in Tippayawat et al. (2023) [30] as follows:

RWC (%) = [(fresh weight − dry weight)/(turgid weight − dry weight)] × 100(3)

#### 4.5.3. Growth and Agronomic Measurements

Yield component traits (stalk height, stalk diameter, internode length, and stalk density) were measured at 1, 3, 6, 8, 10, and 12 MAT/MAH. The stalk height was measured as the distance from the base of the stem at ground level to the top visible dewlap (TVD) using four representative main stalks from a random sample of four stools per subplot. The stalk diameter was measured using a digital vernier caliper from the middle part (lengthwise, avoiding nodes) of four main stalks of the same four stools as above. Stalk density was estimated by counting all millable stalks of the same four stools within the subplot.

#### 4.5.4. Biomass and Leaf Area Index (LAI)

At 1, 3, 6, 8, 10, and 12 MAT/MAH, three representative stools in an area of 2.25 m^2^ were randomly sampled from the center row of each subplot. The plants were cut at the soil surface and partitioned into stalks, leaf blades, leaf sheaths, and dry leaves. Then, the fresh weight of each component was recorded before taking a subsample of 10% by weight of each component for drying. The subsamples were dried at 70 °C for 72 h or until constant weight was achieved. The dry mass of each component was recorded, and the sum of all components was taken as the total biomass per unit area.

Before drying the 10% leaf samples, the total leaf area for each genotype was measured using a leaf area meter (LI-3100, LI-COR Biosciences, Lincoln, NE, USA). The leaf area index (LAI) was expressed as the ratio of leaf area (LA) (cm^2^) and ground area (cm^2^) from which the original samples were collected [30].

The crop growth rate (CGR) was calculated from the total biomass measured over time based on Abu-Ellail et al. (2020) [40] and Sulistiono et al. (2017) [20], as follows:

CGR = (W2 − W1)/(dT)(4)

where W2 and W1 represent the dry weight of the second and first measurements, respectively and dT represents the time interval between the two measurements dates. The height growth rate (HGR) was also calculated for different growth intervals between 1 and 12 MAT/MAH, with Khonghintaisong et al.’s (2021) [28] formula using the stalk height data described above as follows:

HGR = (H2 − H1)/(dT)(5)

where H2 and H1 represent the height of the second and first height measurements, respectively and dT represents the time interval between the two measurements dates.

At 12 MAT/MAH, the cane yield (tons ha^−1^) was estimated by sampling the millable stalks within a designated representative area (33 m^2^) from the two central rows. All millable stalks were harvested at ground level, weighed, and the data extrapolated to metric tons per hectare (tons ha^−1^). Cane yield was used to estimate the drought tolerance index (DTI) for each genotype as per Hoang et al. (2019) [42]:

DTI = Cane yield of stress treatment/Cane yield of non-stress treatment(6)

### 4.6. Statistical Analysis

Data were subjected to analysis of variance, using Statistix ver. 10 [52] based on the split-plot arrangement in a randomized complete block design [53]. The interactive and main effects of water treatment and genotype on the response variables were analyzed with a two-factor analysis of variance (ANOVA). Drought effects were tested with the main plot experimental error (error a). In contrast, the significance of the subplot and the interaction effects were tested with the subplot experimental error (error b) [53]. The least significant difference (LSD) test was used to compare means at the 95% probability level. A test of the homogeneity of the error variances for all traits in the combined analysis of variance over the two crops led to the data for each crop being analyzed separately. The means were compared by using the least significant difference (LSD) at the 95% probability level. Pearson correlation coefficient analysis was performed to determine relationships among traits.

## 5. Conclusions

Drought reduced cane yield and all of its associated traits, with a greater reduction occuring in the long-term compared with the short-term drought treatment. A significant variability in cane yield due to genotypic differences in sensitivity to drought was observed. Strong relationships between cane yield and several yield traits were also observed. Traits such as stalk height, stalk density, internode length, and stalk elongation rates responded to water deficit stress in a manner consistent with their observed yields, indicating their utility for detecting genotypic differences in sensitivity to drought. Stalk diameter proved to be unreliable as a surrogate trait for detecting drought sensitivity, while the HGR, a relatively less cumbersome trait to measure compared with the CGR, proved more effective in predicting cane yield in both the drought and recovery periods.. The early rapid stalk elongation rates of F03–362 and the enhanced dedication of biomass fractions to stalks under the water treatments suggest that this genotype could be exploited in production regions prone to drought or in those that receive ample rainfall. However, its utility in a commercial program would be as a parent for developing new cultivars with a high yield potential under drought stress. This genotype consistently displayed a highly efficient use of water under all water supply regimes, including long-term drought. Its high stalk density, high stalk elongation rate, tall stature, and moderate LAI seem to confer a high cane yield potential. The LAI of F03–362 was also relatively insensitive to drought stress, especially in the planted cane. LAI is a critical trait that is needed to support growth and productivity by intercepting light for photo assimilation. However, genotypes with excessive leaf area production and a high LAI may take up and transpire available moisture inefficiently. This may be the case with genotypes like KK3, which invested a significant amount of resources in LAI development but had cane yields either lower or no greater than those of the other genotypes. Another genotype, TPJ04–768, with a low yield potential under well-watered conditions somehow displayed drought resilience with a consistently low yield reduction under drought (high DTI). Such genotypes are ideal for drought-prone environments as they offer stable yield performance, but unlike F03–362, they are ill-equipped to take advantage of incidental rainfall. TPJ04–768 could have utility in a commercial variety development program because when crossed with high-yield commercially adapted cultivars, it could create segregating populations with progenies combining a high yield potential with a low yield reduction. A deeper understanding of the genetic bases and inheritance of traits like height elongation rates, LAI, stalk density, low yield reduction, and biomass allocation under drought stress will be necessary for breeding for drought resistance and productivity in rainfed cropping systems.

## Figures and Tables

**Table 1:** Mean squares from combined analysis of cane yield and yield components at 12 MAT/MAH (at harvest) for six sugarcane genotypes under three water treatments in the planted cane 2020/21 and the ratoon cane 2021/22.

Source of Variance	DF	Cane Yield	Stalk Height	Stalk Diameter	Stalk Density
		(Tons ha^−1^)	(cm)	(cm)	(Number of Millable Stalks ha^−1^)
Crop year (Y)	1	62,312.6 ** (52.8) ^a^	132,074.0 ** (12.7)	0.1 ^ns^ (0.4)	1.02 × 10^8 ns^ (0.1)
Rep within year	6	171.2 (0.9)	414.0 (0.2)	0.1 (0.7)	7.05 × 10^7^ (0.3)
Water treatment (W)	2	10,418.0 ** (17.6)	59,631.0 ** (11.4)	0.9 ** (4.8)	3.32 × 10^9^ ** (4.1)
W × Y	2	129.6 ^ns^ (0.2)	278.0 ^ns^ (0.1)	0.0 ^ns^ (0.2)	5.25 × 10^7 ns^ (0.1)
Pooled error (a)	12	68.8 (0.7)	331.0 (0.4)	0.5 (2.7)	7.02 × 10^7^ (0.5)
Genotype (G)	5	4127.2 ** (17.5)	144,639.0 ** (69.3)	14.0 ** (78.1)	2.92 × 10^10^ ** (89.5)
G × Y	5	566.6 ** (2.4)	2846.0 ** (1.4)	0.0 ^ns^ (0.1)	1.89 × 10^8^ ** (0.6)
G × W	10	110.8 ^ns^ (0.9)	2074.0 ** (2.0)	0.4 * (2.2)	3.78 × 10^8^ ** (2.3)
G ×W × Y	10	81.9 ^ns^ (0.7)	926.0 ** (0.9)	0.1 ^ns^ (0.5)	4.44 × 10^7 ns^ (0.3)
Pooled error (b)	90	82.9 (6.3)	190.0 (1.6)	1.8 (10.2)	4.23 × 10^7^ (2.3)

Note: DF = degree of freedom; MAT = month after transplanting; MAH = month after harvesting. ns, *, ** = non-significant, significant, and highly significant at *p* ≤ 0.05 and *p* ≤ 0.01 probability level, respectively.

**Table 2:** Pearson correlation coefficients (n = 18) among cane yield (measured at harvest, 12 months) and selected yield components (leaf area index, leaf relative water content, stalk density, stalk height, stalk diameter, stalk internode length, height elongation rate, and crop growth rates measured after cessation of drought treatments, 6 months) of six sugarcane genotypes.

Planted Cane	Cane Yield	LAI	RWC	Stalk Density	Height	Diameter	Internode Length	HGR	CGR
Cane yield_12 MAT	1.000								
LAI_6 MAT	0.808 **	1.000							
RWC_6 MAT	0.107 ^ns^	0.254 ^ns^	1.000						
Stalk density_6 MAT	0.497 *	0.575 *	−0.364 ^ns^	1.000					
Stalk height _6 MAT	0.828 **	0.831 **	−0.006 ^ns^	0.834 **	1.000				
Stalk diameter _6 MAT	−0.059 ^ns^	−0.277 ^ns^	0.501 *	−0.798 **	−0.466 ^ns^	1.000			
Internode length_6 MAT	0.788 **	0.703 **	−0.203 ^ns^	0.752 **	0.890 **	−0.453 ^ns^	1.000		
HGR_(3–6 MAT)	0.863 **	0.870 **	0.157 ^ns^	0.719 **	0.972 **	−0.307 ^ns^	0.865 **	1.000	
CGR_(3–6 MAT)	0.754 **	0.905 **	0.387 ^ns^	0.437 ^ns^	0.805 **	−0.183 ^ns^	0.664 **	0.859 **	1.000
**Ratoon cane**									
Cane yield_12 MAH	1.000								
LAI_6 MAH	0.805 **	1.000							
RWC_6 MAH	0.362 ^ns^	0.363 ^ns^	1.000						
Stalk density_6 MAH	0.792 **	0.678 **	−0.090 ^ns^	1.000					
Stalk height_6 MAH	0.932 **	0.881 **	0.437 ^ns^	0.767 **	1.000				
Stalk diameter_6 MAH	−0.439 ^ns^	−0.467 ^ns^	0.506 *	−0.811 **	−0.446 ^ns^	1.000			
Internode length_6 MAH	0.933 **	0.789 **	0.329 ^ns^	0.797 **	0.957 **	−0.516 *	1.000		
HGR_(3–6 MAH)	0.889 **	0.828 **	0.473 ^ns^	0.677 **	0.969 **	−0.331 ^ns^	0.946 **	1.000	
CGR_(3–6 MAH)	0.761 **	0.872 **	0.643 **	0.506 *	0.840 **	−0.166 ^ns^	0.757 **	0.831 **	1.000

**Table 3:** Mean square from combined analysis of variance for height growth rate (HGR) of six sugarcane genotypes before, during, and after three drought treatment durations over two growing seasons (crop year; planted cane, 2020/21, and ratoon cane, 2021/22).

Source of Variance	DF	Height Growth Rate (HGR) (cm day^−1^)				
		Drought Period		Recovery Period		Physiological Maturity
		1–3 Months	3–6 Months	6–8 Months	8–10 Months	10–12 Months
Crop year (Y)	1	0.17 ** (1.6) ^a^	3.50 ** (11.2)	7.98 ** (30.9)	7.12 ** (29.8)	13.45 ** (25.7)
Rep within year	6	0.00 (0.1)	0.04 (0.8)	0.13 (2.9)	0.06 (1.6)	0.07 (0.9)
Water treatment (W)	2	1.24 ** (23.4)	7.17 ** (45.9)	0.08 ^ns^ (0.6)	0.80 ** (6.7)	0.00 ^ns^ (0.0)
W × Y	2	0.22 ** (4.1)	0.12 ^ns^ (0.7)	0.28 ^ns^ (2.2)	0.39 * (3.3)	0.94 ** (3.6)
Pooled error (a)	12	0.00 (0.5)	0.03 (1.3)	0.11 (5.1)	0.10 (4.9)	0.06 (1.3)
Genotype (G)	5	1.21 ** (57.5)	1.77 ** (28.4)	1.12 ** (21.7)	0.55 ** (11.4)	3.23 ** (30.8)
G × Y	5	0.01 ** (0.6)	0.09 ** (1.5)	0.18 ** (3.5)	0.09 ^ns^ (1.9)	0.24 ** (2.3)
G × W	10	0.09 ** (8.6)	0.12 ** (3.9)	0.10 ^ns^ (4.0)	0.25 ** (10.7)	1.07 ** (20.4)
G ×W × Y	10	0.01 ** (1.3)	0.09 ** (2.7)	0.23 ** (8.7)	0.10 ^ns^ (4.1)	0.22 ** (4.2)
Pooled error (b)	90	0.00 (2.2)	0.01 (3.4)	0.06 (20.4)	0.07 (25.6)	0.06 (10.9)

Note: DF = degrees of freedom; ns, *, ** = non-significant, significant, and highly significant at *p* ≤ 0.05 and *p* ≤ 0.01 probability level, respectively.

**Table 4:** Mean of height growth rate (HGR) (cm day^−1^) of sugarcane genotypes under three water regimes. During each crop year (planted cane, 2020/21 or ratoon cane 2021/22), rates were measured before (1–3 MAT/MAH), during (3–6 MAT/MAH), and after (6–8 MAT/MAH) drought treatments.

Planted Cane	HGR at 1–3 MAT			HGR at 3–6 MAT			HGR at 6–8 MAT		
Genotypes	SD0	SD1	SD2	SD0	SD1	SD2	SD0	SD1	SD2
F03–362	0.98 ± 0.03 Aa	0.98 ± 0.03 Aa	0.45 ± 0.03 Ba	2.32 ± 0.08 Aa	1.62 ± 0.07 Ba	1.71 ± 0.07 Ba	1.77 ± 0.23 Ba	2.00 ± 0.11 ABabc	2.28 ± 0.05 Aab
KK09–0358	0.41 ± 0.02 Ab	0.41 ± 0.04 Ab	0.26 ± 0.03 Bb	2.01 ± 0.07 Ab	1.31 ± 0.06 Bb	0.95 ± 0.05 Cb	2.07 ± 0.12 Aa	2.35 ± 0.11 Aa	2.38 ± 0.17 Aa
KK09–0939	0.40 ± 0.04 Ab	0.39 ± 0.03 Ab	0.13 ± 0.02 Bc	1.76 ± 0.09 Ac	1.25 ± 0.09 Bbc	0.73 ± 0.06 Cc	2.21 ± 0.23 Aa	2.14 ± 0.01 Aab	1.77 ± 0.17 ABc
TPJ04–768	0.21 ± 0.03 Ac	0.18 ± 0.01 Ad	0.10 ± 0.03 Bc	1.70 ± 0.07 Acd	1.21 ± 0.09 Bbc	0.69 ± 0.01 Cc	2.20 ± 0.137 Aa	1.60 ± 0.13 Bcd	2.12 ± 0.19 ABabc
KK3	0.22 ± 0.01 Ac	0.20 ± 0.03 Acd	0.16 ± 0.03 Ac	1.58 ± 0.10 Acd	1.16 ± 0.07 Bc	1.00 ± 0.06 Bb	1.81 ± 0.25 Aa	1.45 ± 0.19 Ad	1.76 ± 0.23 Ac
UT12	0.24 ± 0.01 Bc	0.28 ± 0.01 Ac	0.13 ± 0.01 Cc	1.46 ± 0.06 Ad	1.24 ± 0.08 Bbc	0.56 ± 0.04 Cd	1.83 ± 0.04 Aa	1.77 ± 0.18 Abcd	1.72 ± 0.23 Ac
Mean	0.41 A	0.41 A	0.20 B	1.81 A	1.30 B	0.94 C	1.98 A	1.88 A	2.00 A
F-test	**	**	**	**	**	**	ns	**	*
**Ratoon cane**	**HGR at 1–3 MAH**			**HGR at 3–6 MAH**			**HGR at 6–8 MAH**		
	**SD0**	**SD1**	**SD2**	**SD0**	**SD1**	**SD2**	**SD0**	**SD1**	**SD2**
F03–362	1.07 ± 0.05 Aa	0.90 ± 0.04 Ba	0.43 ± 0.02 Ca	1.78 ± 0.05 Aa	1.25 ± 0.03 Ba	1.37 ± 0.07 Ba	1.87 ± 0.07 ABa	1.65 ± 0.06 Ba	1.98 ± 0.06 Aa
KK09–0358	0.78 ± 0.04 Ab	0.37 ± 0.04 Bbc	0.25 ± 0.02 Cb	1.43 ± 0.04 Ab	1.25 ± 0.06 Ba	0.94 ± 0.04 Cb	1.65 ± 0.05 Aab	1.81 ± 0.07 Aa	1.51 ± 0.09 Abc
KK09–0939	0.80 ± 0.03 Ab	0.39 ± 0.03 Bb	0.10 ± 0.01 Cc	1.34 ± 0.03 Abc	1.01 ± 0.07 Bb	0.45 ± 0.03 Cc	1.43 ± 0.07 Ab	1.70 ± 0.08 Aa	1.57 ± 0.07 Ab
TPJ04–768	0.45 ± 0.02 Ac	0.18 ± 0.03 Be	0.10 ± 0.03 Cc	1.32 ± 0.07 Abc	1.00 ± 0.06 Bb	0.92 ± 0.08 Bb	1.15 ± 0.09 Cc	1.75 ± 0.03 Aa	1.36 ± 0.03 Bc
KK3	0.38 ± 0.03 Acd	0.24 ± 0.03 Bde	0.20 ± 0.01 Bb	1.27 ± 0.07 Ac	0.68 ± 0.07 Bc	0.41 ± 0.01 Ccd	1.07 ± 0.13 Bc	1.33 ± 0.01 Ab	1.44 ± 0.03 Abc
UT12	0.32 ± 0.01 Ad	0.28 ± 0.02 Bcd	0.13 ± 0.02 Cc	1.26 ± 0.08 Ac	0.69 ± 0.03 Bc	0.28 ± 0.04 Cd	1.05 ± 0.13 Ac	1.20 ± 0.04 Ab	1.18 ± 0.04 Ad
Mean	0.63 A	0.39 B	0.20 C	1.40 A	0.98 B	0.73 C	1.37 B	1.57 A	1.51 AB
F-test	**	**	**	**	**	**	**	**	**

Note: SD0 = no water stress, SD1 = short-term water treatment, and SD2 = long-term water treatment. Different uppercase letters across a row indicate significant differences among irrigation treatments and different lowercase letters along a column indicate significant differences among genotypes by LSD test at *p* ≤ 0.05. Values are mean ± SE (n = 4). The F-test across rows shows the differences among irrigation treatments when averaged across all genotypes and compared within sampling periods (1–3 MAT/MAH; 3–6 MAT/MAH; 6–8 MAT/MAH). In this test, ns, *, ** indicates non-significant, significant, and highly significant at *p* ≤ 0.05 and *p* ≤ 0.01 probability levels, respectively.

**Table 5:** Mean square from combined analysis of variance for crop growth rate (CGR) of six sugarcane genotypes before, during, and after three drought treatment durations over two growing seasons (crop year; planted cane, 2020/21, and ratoon cane, 2021/22).

Source of Variance	DF	Crop Growth Rate (CGR) (g m^−2^ day^−1^)				
		Drought Period		Recovery Period		Physiological Maturity
		1–3 Months	3–6 Months	6–8 Months	8–10 Months	10–12 Months
Crop year (Y)	1	0.05 ** (27.5) ^a^	0.05 ** (8.0)	0.33 ** (27.3)	1.87 ** (25.8)	0.00 ^ns^ (0.0)
Rep within year	6	0.00 (0.4)	0.00 (1.2)	0.00 (2.5)	0.00 (0.2)	0.00 (0.8)
Water treatment (W)	2	0.02 ** (22.1)	0.22 ** (65.6)	0.02 * (4.2)	1.45 ** (40.3)	0.03 * (2.5)
W × Y	2	0.02 ** (19.3)	0.00 ** (1.2)	0.03 ** (5.0)	0.02 ^ns^ (0.5)	0.04 ** (3.2)
Pooled error (a)	12	0.00 (0.6)	0.00 (0.2)	0.00 (4.3)	0.01 (1.2)	0.01 (3.1)
Genotype (G)	5	0.01 ** (18.3)	0.02 ** (13.7)	0.03 ** (13.6)	0.12 ** (8.5)	0.11 ** (21.1)
G × Y	5	0.00 ** (3.3)	0.00 ^ns^ (0.2)	0.01 ** (5.8)	0.01 * (0.8)	0.05 ** (9.8)
G × W	10	0.00 ** (2.4)	0.00 ** (3.2)	0.02 ** (14.1)	0.11 ** (15.7)	0.05 ** (18.3)
G × W × Y	10	0.00 ** (1.7)	0.00 ** (2.4)	0.01** (8.9)	0.01 ** (1.7)	0.01 ^ns^ (3.8)
Pooled error (b)	90	0.00 (4.4)	0.00 (4.3)	0.00 (14.4)	0.00 (5.4)	0.01 (37.4)

Note: DF = degrees of freedom; ns, *, ** = non-significant, significant, and highly significant at *p* ≤ 0.05 and *p* ≤ 0.01 probability level, respectively.

**Table 6:** Mean crop growth rate (CGR) (cm day^−1^) of sugarcane genotypes under three water regimes. During each crop year (planted cane, 2020/21 or ratoon cane 2021/22), rates were measured before (1–3 MAT/MAH), during (3–6 MAT/MAH), and after (6–8 MAT/MAH) drought treatments.

Planted Cane	CGR at 1–3 MAT			CGR at 3–6 MAT			CGR at 6–8 MAT		
Genotypes	SD0	SD1	SD2	SD0	SD1	SD2	SD0	SD1	SD2
F03–362	0.068 ± 0.003 Aab	0.064 ± 0.001 Ba	0.051 ± 0.004 Ca	0.304 ± 0.011 Aa	0.192 ± 0.009 Ba	0.129 ± 0.003 Cab	0.237 ± 0.035 Bc	0.330 ± 0.046 ABa	0.386 ± 0.024 Ba
KK09–0358	0.064 ± 0.004 Ab	0.050 ± 0.003 Abc	0.036 ± 0.002 Bb	0.285 ± 0.013 Aab	0.174 ± 0.003 Bab	0.110 ± 0.014 Cb	0.291 ± 0.019 ABb	0.351 ± 0.032 Aa	0.259 ± 0.028 Bb
KK09–0939	0.068 ± 0.002 Aab	0.048 ± 0.002 Abc	0.035 ± 0.003 Bbc	0.267 ± 0.016 Ab	0.185 ± 0.007 Ba	0.120 ± 0.009 Cab	0.280 ± 0.015 Abc	0.247 ± 0.029 Ab	0.237 ± 0.023 Abc
TPJ04–768	0.083 ± 0.003 Aa	0.051 ± 0.003 Bb	0.030 ± 0.004 Cbc	0.266 ± 0.009 Ab	0.185 ± 0.011 Ba	0.134 ± 0.004 Ca	0.263 ± 0.013 Abc	0.291 ± 0.035 Aab	0.121 ± 0.025 Bd
KK3	0.061 ± 0.001 Ab	0.036 ± 0.001 Ad	0.026 ± 0.002 Bc	0.221 ± 0.013 Ac	0.136 ± 0.013 Bc	0.065 ± 0.007 Cc	0.405 ± 0.024 Aa	0.294 ± 0.027 Bab	0.210 ± 0.012 Cbc
UT12	0.075 ± 0.003 Aab	0.044 ± 0.003 Ac	0.038 ± 0.002 Ab	0.208 ± 0.007 Ac	0.151 ± 0.012 Bbc	0.073 ± 0.008 Cc	0.146 ± 0.013 Ad	0.223 ± 0.015 Bb	0.187 ± 0.008 Ccd
Mean	0.053 A	0.049 A	0.036 B	0.259 A	0.170 B	0.105 C	0.270 AB	0.289 A	0.233 B
F-test	**	**	**	**	**	**	**	*	**
**Ratoon cane**	**CGR at 1–3 MAH**			**CGR at 3–6 MAH**			**CGR at 6–8 MAH**		
	**SD0**	**SD1**	**SD2**	**SD0**	**SD1**	**SD2**	**SD0**	**SD1**	**SD2**
F03–362	0.154 ± 0.008 Aa	0.125 ± 0.002 Ba	0.070 ± 0.007 Ca	0.236 ± 0.015 Aa	0.173 ± 0.007 Ba	0.126 ± 0.011 Ca	0.263 ± 0.013 Aab	0.142 ± 0.022 Bbc	0.250 ± 0.017 Aa
KK09–0358	0.123 ± 0.007 Ab	0.118 ± 0.009 Aa	0.067 ± 0.004 Ba	0.238 ± 0.003 Aa	0.100 ± 0.008 Bb	0.091 ± 0.007 Bb	0.206 ± 0.020 Abc	0.192 ± 0.013 Aa	0.173 ± 0.021 Ab
KK09–0939	0.102 ± 0.002 Ac	0.115 ± 0.009 Aa	0.031 ± 0.003 Bb	0.188 ± 0.010 Ab	0.183 ± 0.015 Aa	0.084 ± 0.005 Bb	0.305 ± 0.037 Aa	0.107 ± 0.007 Bc	0.121 ± 0.038 Bb
TPJ04–768	0.108 ± 0.004 Abc	0.107 ± 0.013 Aa	0.033 ± 0.002 Bb	0.185 ± 0.002 Ab	0.171 ± 0.005 Aa	0.088 ± 0.005 Bb	0.189 ± 0.018 Ac	0.098 ± 0.024 Bc	0.104 ± 0.012 Bb
KK3	0.063 ± 0.007 Ad	0.061 ± 0.004 Ab	0.027 ± 0.003 Bb	0.194 ± 0.008 Ab	0.095 ± 0.004 Bb	0.041 ± 0.002 Cc	0.145 ± 0.026 Ac	0.107 ± 0.015 Ac	0.149 ± 0.025 Ab
UT12	0.113 ± 0.001 Abc	0.110 ± 0.003 Aa	0.025 ± 0.001 Bb	0.167 ± 0.008 Ab	0.074 ± 0.009 Bb	0.073 ± 0.012 Bb	0.161 ± 0.007 Ac	0.176 ± 0.017 Aab	0.154 ± 0.042 Ab
Mean	0.111 A	0.106 A	0.042 B	0.201 A	0.133 B	0.084 C	0.212 A	0.137 B	0.158 B
F-test	**	**	**	**	**	**	**	**	**

Note: SD0 = no water stress, SD1 = short-term water treatment, and SD2 = long-term water treatment. Different uppercase letters across a row indicate significant differences among irrigation treatments and different lowercase letters along a column indicate significant differences among genotypes by LSD test at *p* ≤ 0.05. Values are mean ± SE (n = 4). The F-test across rows shows the differences among irrigation treatments averaged across all genotypes and compared within sampling periods (1–3 MAT/MAH; 3–6 MAT/MAH; 6–8 MAT/MAH). In this test, ns, *, ** indicates non-significant, significant, and highly significant at *p* ≤ 0.05 and *p* ≤ 0.01 probability levels, respectively.

**Table 7:** The six sugarcane genotypes used in the experiment, their pedigree, distinguishing characteristics, and sources of origin.

Genotypes	Parents	Sources	Characteristics ^#^
F03–362 (F_1_)	88–2–401 × ThS98–178 + ThS98–264 (bulk pollen)	KKFCRC *	High biomass and fiber, low sugar yield
KK09–0358 (BC_1_)	95–2–317 × F03–381 (F_1_)	KKFCRC *	High cane yield, medium sugar yield
KK09–0939 (BC_2_)	BC04–251 (BC_1_) × UT4	KKFCRC *	High cane yield and sugar yield
TPJ04–768 (BC_1_)	94–2–128 × F03–331 (F_1_)	KKFCRC *	High cane yield, medium sugar yield
KK3	85–2–352 × K84–200	KKFCRC *	Check (early drought tolerant) high cane yield and high sugar content
UT12	SP80 × UT3	SPFCRC **	Check (drought susceptible) and high cane yield under irrigated conditions

* KKFCRC = Khon Kaen Field Crops Research Center, Thailand; ** SPFCRC = Suphan Buri Field Crops Research Center, Thailand; ^#^ Khonghintaisong et al., 2018; 2021 [28]; Chumphu et al., 2019 [38]; Set-Tow et al., 2020 [44]; Leanasawat et al., 2021 [5]; Jumkudling et al., 2022 [45].

## Data Availability

The data presented in this study are available within the article/Supplementary Materials.

## Supplementary Materials

The following supporting information can be downloaded at https://www.mdpi.com/article/10.3390/plants14050796/s1, Figure S1: Monthly total rainfall (mm), monthly average maximum temperature (°C), minimum temperature (°C), solar radiation (MJ m^−2^ day^−1^), relative humidity (%), and evaporation (mm) during the trial period of the planted cane 2020/21 (a) and in the ratoon cane 2021/22 (b) under no water stress (SD0), SD1 = short-term water treatment at 3–6 months after transplanting (MAT) in the planted cane and short-term water treatment at 3–6 months after harvesting (MAH) in the ratoon cane, SD2 = long-term water treatment at 1–6 MAT/MAH. Water was reintroduced during the recovery period (6–10 MAT/MAH) while the maturity period (10–12 MAT/MAH) is a phenological stage when sugarcane undergoes ripening. Figure S2: Average of Leaf relative water content (RWC) at 1, 3, 4, 5, 6, 8, and 10 months after transplanting (MAT) in the planted cane 2020/21 (a) and at 1, 3, 6, 8, and 10 months after harvesting (MAH) in the ratoon cane 2021/22 (b) of six sugarcane genotypes under no water stress (SD0), short-term water treatment (SD1) and long-term water treatment (SD2) of sugarcane field. Values are mean ± SE (n = 24). Water was reintroduced during the recovery period (6–10 MAT/MAH) while the maturity period (10–12 MAT/MAH) is a phenological stage when sugarcane undergoes ripening. Table S1: Physical and chemical properties of the soil in the experimental field at depths of 0–30 cm and 30–60 cm.
