# Supplementary material for: Timing and Duration of Drought Differentially Affect Growth and Yield Components Among Sugarcane Genotypes
Source: Plants (Basel). 2025 Mar 4;14(5):796. doi: 10.3390/plants14050796 (PMC11902036; doi:10.3390/plants14050796)
Supplement: Supplementary file 1 [file plants-14-00796-s001.zip › plants-3397948-supplementary.pdf]

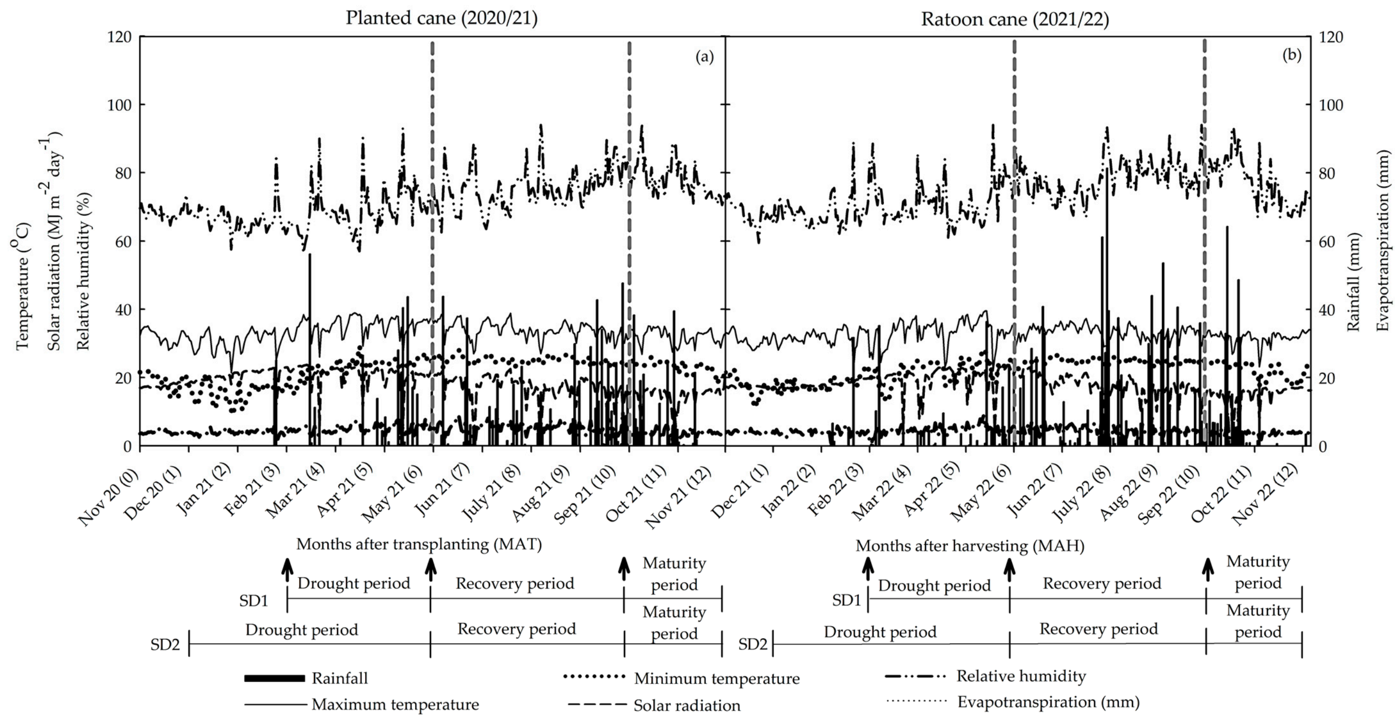

Figure S1. Monthly total rainfall (mm), monthly average maximum temperature (°C), minimum temperature (°C), solar radiation (MJ m^{-2} day^{-1}), relative humidity (%), and evaporation (mm) during the trial period of the planted cane 2020/21 (a) and in the ratoon cane 2021/22 (b) under no water stress (SD0), SD1 = short–term water treatment at 3–6 months after transplanting (MAT) in the plant cane and short–term water treatment at 3–6 months after harvesting (MAH) in the ratoon cane, SD2 = long–term water treatment at 1–6 MAT/MAH. Water was reintroduced during the recovery period (6-10 MAT/MAH) while the maturity period (10-12 MAT/MAH) is a phenological stage when sugarcane undergoes ripening.

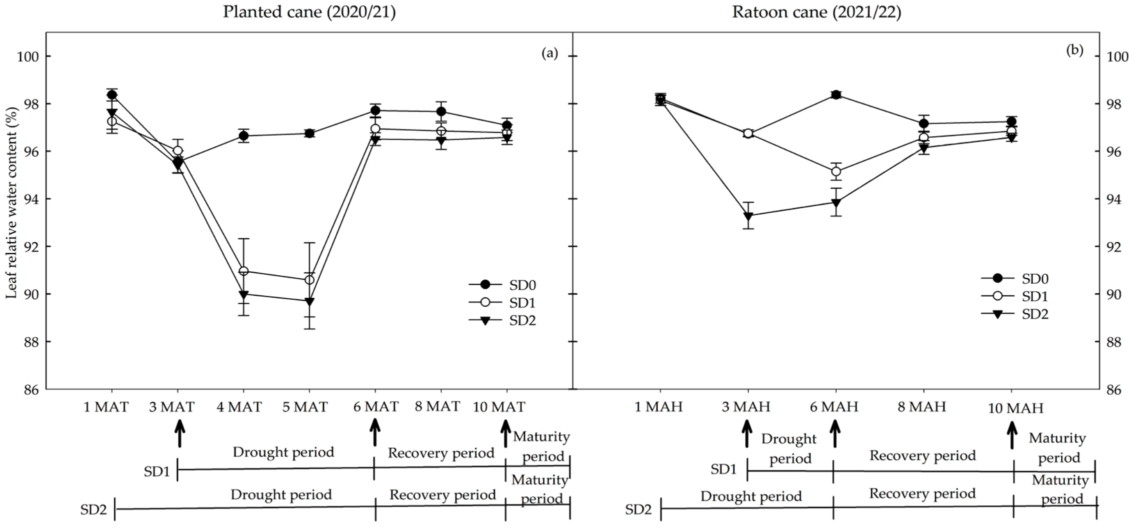

Figure S2. Average of leaf relative water content (RWC) at 1, 3, 4, 5, 6, 8, and 10 months after transplanting (MAT) in the planted cane 2020/21 (a) and at 1, 3, 6, 8, and 10 months after harvesting (MAH) in the ratoon cane 2021/22 (b) of six sugarcane genotypes under no water stress (SD0), short–term water treatment (SD1) and long–term water treatment (SD2) of sugarcane field. Values are mean ± SE (n = 24). Water was reintroduced during the recovery period (6-10 MAT/MAH) while the maturity period (10-12 MAT/MAH) is a phenological stage when sugarcane undergoes ripening.

Table S1. Physical and chemical properties of the soil in the experimental field at depths of 0-30 cm and 30-60 cm.

Soil depth [cm]	pH [1:1 H_2O]	EC [1:5 H_2O] dS m^{-1}	OM [%]	Available P [mg kg^{-1}]	Exchangeable			Bulk density	Texture
					K [mg kg^{-1}]	Ca [mg kg^{-1}]	Mg [mg kg^{-1}]		
0-30	5.35	0.0138	0.29	51.82	46.04	77.59	8.39	1.55	Sandy loam
30-60	5.83	0.0170	0.13	53.16	51.56	158.43	15.79	1.70	Sandy loam

EC= electrical conductivity; OM=organic matter
